# Histone Deacetylase 6 and the Disease Mechanisms of α-Synucleinopathies

**DOI:** 10.3389/fnsyn.2020.586453

**Published:** 2020-09-11

**Authors:** Miguel Lemos, Nadia Stefanova

**Affiliations:** Department of Neurology, Medical University of Innsbruck, Innsbruck, Austria

**Keywords:** histone deacetylase 6, α-synuclein, α-synucleinopathies, Parkinson’s disease, multiple system atrophy, neurodegeneration, protein degradation

## Abstract

The abnormal accumulation of α-Synuclein (α-Syn) is a prominent pathological feature in a group of diseases called α-Synucleinopathies, such as Parkinson’s disease, dementia with Lewy bodies (DLB), and multiple system atrophy (MSA). The formation of Lewy bodies (LBs) and glial cytoplasmic inclusions (GCIs) in neurons and oligodendrocytes, respectively, is highly investigated. However, the molecular mechanisms behind α-Syn improper folding and aggregation remain unclear. Histone deacetylase 6 (HDAC6) is a Class II deacetylase, containing two active catalytic domains and a ubiquitin-binding domain. The properties of HDAC6 and its exclusive cytoplasmic localization allow HDAC6 to modulate the microtubule dynamics, acting as a specific α-tubulin deacetylase. Also, HDAC6 can bind ubiquitinated proteins, facilitating the formation of the aggresome, a cellular defense mechanism to cope with higher levels of misfolded proteins. Several studies report that the aggresome shares similarities in size and composition with LBs and GCIs. HDAC6 is found to co-localize with α-Syn in neurons and in oligodendrocytes, together with other aggresome-related proteins. The involvement of HDAC6 in several neurodegenerative diseases is already under discussion, however, the results obtained by modulating HDAC6 activity are not entirely conclusive. The main goal of this review is to summarize and critically discuss previous *in vitro* and *in vivo* data regarding the specific role of HDAC6 in the context of α-Syn accumulation and protein aggregation in α-Synucleinopathies.

## Introduction

Protein misfolding and consequent accumulation is a biological process that has been intensively studied for many years. To perform their function within the cell, the newly synthesized proteins must achieve the proper conformation in a three-dimensional structure. However, due to disturbances in the folding process, such as environmental insults or genetic mutations, certain proteins may fail to achieve the native conformation (Balchin et al., 2016). Through a mechanism that is still not well understood, those proteins can undergo a process of misfolding and aggregation, leading to the formation of neurotoxic inclusions, a feature of age-related neurodegenerative diseases (Ross and Poirier, 2004; Sweeney et al., 2017; Soto and Pritzkow, 2018).

In this review article, we focus exclusively on the process of α-synuclein (α-Syn) accumulation and aggregation, the pathological hallmark of a group of diseases like Parkinson’s disease (PD), multiple system atrophy (MSA) and dementia with Lewy bodies (DLB), commonly known as α-Synucleinopathies (Duda et al., 2000; Goedert et al., 2017). To date, it is well-accepted that α-Syn is the major component of pathological aggregates, such as Lewy bodies (LBs), Lewy neurites (LNs), and glial cytoplasmic inclusions (GCIs; Spillantini and Goedert, 2000; Visanji et al., 2019). It is believed that these protein inclusions play a central role in the progression of the disease, contributing to neuronal dysfunction, neuroinflammation, and neurodegeneration (Vekrellis et al., 2011; Uchihara and Giasson, 2016). However, the molecular mechanisms involved in the formation of such inclusions are not fully understood.

To cope with higher amounts of pathological α-Syn aggregates, several protein quality control mechanisms are triggered to maintain the normal function and homeostasis of the neural cells (Ciechanover and Kwon, 2015). However, in a disease context, the exacerbated amount of pathological α-Syn leads to the dysfunction of the ubiquitin-proteasome system (UPS), resulting in a higher accumulation of toxic aggregates (Ciechanover and Kwon, 2015; Kaushik and Cuervo, 2015; da Fonseca et al., 2015; Zheng et al., 2016). As a consequence, the aggresome-autophagy pathway is stimulated, in which misfolded and aggregated proteins are transported to a perinuclear aggresome (Olzmann et al., 2008). Interestingly, LBs and aggresomes share several biochemical and morphological characteristics (Olanow et al., 2004; Miki et al., 2011).

One important regulator involved in the formation of the aggresome is the histone deacetylase 6 (HDAC6), by linking ubiquitinated proteins to the microtubule dynein motor complex (Boyault et al., 2007a,b; Ouyang et al., 2012). HDAC6 is a protein from the histone deacetylase superfamily, containing two active catalytic domains (Yang and Grégoire, 2005). It is mainly localized in the cytoplasm and mediates the deacetylation of non-histone proteins, more specifically α-tubulin, heat-shock protein 90 (HSP90) and cortactin, thus being involved in the regulation of the microtubule dynamics and in the transfer of misfolded proteins to the aggresome (Simões-Pires et al., 2013). In α-Synucleinopathies, HDAC6 co-localizes with α-Syn in LBs and GCIs from PD and MSA samples, respectively (Miki et al., 2011).

In this review article, we aim to highlight previous findings regarding α-Syn aggregation, together with *in vivo* and *in vitro* data focusing on the specific role of HDAC6 in the formation of such protein inclusions. These studies may open new avenues towards a promising therapeutic target in α-Synucleinopathies.

## α-Synuclein: An Enigmatic Protein

α-Syn is a protein with 14 kDa (containing 140 amino acids residues), mostly found in the nucleus and in the presynaptic terminals of the central nervous system (CNS; Jakes et al., 1994). It is composed of three distinct regions: (1) an amino terminus containing lipid-binding motif, which allows membrane binding and facilitates the formation of α-helical structures; (2) a central hydrophobic region, also known as the non-Amyloid β component (NAC); and (3), an unstructured and negatively charged carboxyl terminus (Mochizuki et al., 2018). α-Syn, together with β- and γ-Synuclein compose the synuclein family, however, what makes α-Syn structurally unique is the presence of the NAC region, which in turn confers the β-sheet forming potential and facilitates its aggregation process (Wong and Krainc, 2017).

Under physiologic conditions, α-Syn is considered an intrinsically unstructured protein, lacking a defined conformation (Theillet et al., 2016). It has been described that α-Syn exists mostly as a monomer, however, due to its structural flexibility, α-Syn can transit between monomeric and oligomeric states, under a highly-balanced process (Lashuel et al., 2013). As a consequence of such conformational flexibility and its localization at the presynaptic terminals, it is believed that α-Syn has multifunctional properties in the CNS, consisting in the regulation of neurotransmitter release, synaptic function and synaptic plasticity (Lashuel et al., 2013; Ghiglieri et al., 2018).

The attention to this protein expanded upon the discovery of a mutation in the *SNCA* gene, associated with early-onset familial forms of PD (Polymeropoulos et al., 1997). Afterward, in 1997, α-Syn was identified as one of the major components of LBs and LNs, neuronal cytoplasmic inclusions considered to be the pathological hallmark of PD and DLB (Spillantini et al., 1997). A year later, α-Syn was confirmed as the main component of GCIs in MSA (Spillantini et al., 1998; Wakabayashi et al., 1998). To date, it is well-accepted that aggregated α-Syn represents a key feature in the pathogenesis of this group of diseases called α-Synucleinopathies (Stefanis, 2012; Visanji et al., 2019).

Mutations in the SNCA gene and the presence of stress-induced conditions within the cell can lead to increased levels of α-Syn and consequent disruption of the equilibrium between monomeric and oligomeric species (Auluck et al., 2010; Wales et al., 2013). Despite the presence of protein homeostasis mechanisms, α-Syn can undergo a process of uncontrolled oligomerization from soluble oligomeric species into large, insoluble fibrils, resulting in the formation of structures like LBs (Stefanis, 2012). The process of α-Syn aggregation can be potentiated in disease-induced stress conditions in the cells, such as changes in the pH, temperature, oxidative stress, mitochondria dysfunction, and post-translational modifications (Hasegawa et al., 2002; Anderson et al., 2006). In the healthy brain, α-Syn homeostasis is promoted by combined actions of molecular chaperones, the UPS, and finally, the lysosome autophagy system (Ghiglieri et al., 2018). On the other hand, the accumulation of α-Syn into proteinaceous inclusions creates a scenario in which those protein quality control mechanisms become impaired, resulting in a vicious cycle that aggravates the neurodegeneration process (Djajadikerta et al., 2020).

Under pathological conditions, the failure of intracellular mechanisms of α-Syn clearance might contribute to the pathological release of toxic α-Syn oligomeric species to neighboring cells (Lee et al., 2013; da Fonseca et al., 2015). Recent findings show that soluble and insoluble α-Syn aggregates can be secreted and propagate through connected neuronal regions, in a stereotypical pattern, such as the hypothesis developed by Braak and colleagues, commonly known as the Braak’s hypothesis for PD (Braak et al., 2003; McCann et al., 2016). This is evidenced by the presence of α-Syn aggregates in several areas of the CNS, and not only in brain regions where the level of neurodegeneration is more accentuated (Rey et al., 2019). Furthermore, these α-Syn aggregates can be transferred between different brain cells, triggering the oligomerization and aggregation of the native monomeric α-Syn species (Luk et al., 2012; Rey et al., 2019). Such pathological characteristic of α-Syn aggregates was observed upon the addition of recombinant α-Syn fibrils in cultured cells, resulting in the recruitment of the endogenous α-Syn and consequent formation of LBs (Luk et al., 2009).

## α-Synucleinopathies

α-Synucleinopathies represent a group of neurodegenerative disorders characterized by the misfolding and aggregation of α-Syn forming LBs and LNs within neurons, or GCIs in oligodendrocytes (Jellinger, 2003; Peelaerts and Baekelandt, 2016). Primary α-Synucleinopathies include diseases such as PD, DLB, and MSA, distinguished by specific clinical and pathological manifestations (Galasko, 2017; Yang and Yu, 2017; Nussbaum, 2018).

PD is the most common motor neurodegenerative disorder, affecting mostly people over the age of 60 years. Clinical manifestations of PD include motor signs such as rigidity, bradykinesia, gait impairment, and resting tremor (Jankovic, 2008; Poewe et al., 2017; Del Rey et al., 2018). In addition to motor symptoms, patients suffering from PD also show several non-motor symptoms, including olfactory dysfunction, REM sleep abnormalities, cognitive impairment, and autonomic failure that may precede the onset of the motor symptoms (Fereshtehnejad et al., 2015; Kalia and Lang, 2015). Pathologically, PD is characterized by filamentous inclusions of α-Syn in neurons, named LBs or Lewy neurites. Also, selective neuronal loss is observed in multiple brain regions that define the clinical presentation of the disease. One of the most affected brain regions is the substantia nigra pars compacta (SNc; Jankovic, 2008; Stefanis, 2012).

In DLB, dementia is accompanied by motor symptoms similar to those in PD, however, the patients with DLB tend to have a poorer response to levodopa treatment (Burn et al., 2003). In most of the cases, autonomic features are also present, such as orthostatic hypotension (Yang and Yu, 2017). Opposed to PD, postmortem analysis of DLB brains reveals less neurodegeneration in the SNc, and an advanced Braak staging in the cerebral cortex and hippocampus, contributing to dementia and Alzheimer’s disease (AD)-related symptoms (Dodel et al., 2008).

MSA is a unique α-Synucleinopathy, different from PD and DLB in several ways. It is characterized by a highly variable combination of Parkinsonism, cerebellar ataxia, and autonomic failure, which makes the correct diagnosis sometimes difficult (Jellinger, 2014; Fanciulli and Wenning, 2015). According to the predominance of the parkinsonian or the cerebellar features, the clinical presentation is divided into two subtypes: the MSA-P, where the striatonigral degeneration is more predominant, and MSA-C, where the olivopontocerebellar projections are mostly affected (Valera and Masliah, 2018). In both cases, the patients also show non-motor symptoms, with the most common being an autonomic failure (Fanciulli and Wenning, 2015). The pathological hallmark of MSA is the presence of aggregated α-Syn in the cytoplasm of oligodendrocytes, forming the so-called GCIs (Jellinger and Lantos, 2010). The source of α-Syn in MSA oligodendrocytes remains debatable to date (Miller et al., 2005; Asi et al., 2014; Djelloul et al., 2015). It is suggested that primary oligodendrogliopathy, α-Syn expression in oligodendroglia, and/or α-Syn uptake from surrounding neurons may play a role in the pathogenesis of MSA (Wenning et al., 2008; Fellner et al., 2011; Stefanova and Wenning, 2016).

## The Histone Deacetylase Superfamily: Why Is HDAC6 Unique?

Histone deacetylases (HDACs) are a group of enzymes that promote the deacetylation of lysine residues of histones, as well as cytoplasmic, nuclear, or mitochondrial proteins (Ruijter et al., 2003). The process of lysine deacetylation in histones has been extensively studied over the past years for its role in the regulation of epigenetic modifications and concerning certain types of cancer (Seidel et al., 2015). However, a growing number of identified non-histone substrates has been demonstrating that such enzymes can also regulate important cellular mechanisms such as cell proliferation, intracellular trafficking, and protein stability (Glozak et al., 2005; Haberland et al., 2011). As a consequence, selective HDAC inhibitors have been developed and contributed for a better understanding of the different functions and properties of several members of the HDAC superfamily (Prince et al., 2009; Dietz and Casaccia, 2010; Thaler and Mercurio, 2014; Didonna and Opal, 2015).

To date, HDAC enzymes are divided into four different classes based on their sequence homology to yeast deacetylases, cellular localization, and specific substrates (Ruijter et al., 2003). Class I HDACs include HDAC 1, 2, 3, and 8, which are ubiquitously expressed in the nucleus thus regulating the transcription of genes. Class II HDACs can shuttle between the cytoplasm and the nucleus and are expressed in specific body tissues. Class II is subdivided into Class IIa (HDAC 4, 5, 7, and 9), and Class IIb (HDAC 6 and 10). Class III HDACs are called Sirtuins. They are NAD^+^-dependent enzymes and show different structural features. Finally, Class IV is composed only of HDAC11, showing similarities with some Class I and II HDACs. Classes I, II, and IV are referred to as “classical HDACs” due to their zinc-dependent catalytic activity (Ruijter et al., 2003).

The activity of HDAC enzymes is opposed to the functions of histone acetyltransferases (HATs; Saha and Pahan, 2006). In a disease context, an imbalance between HDACs and HATs has been described affecting histone deacetylation and the transcription of genes involved in neuroprotection and apoptosis (Saha and Pahan, 2006). The role of each HDAC enzyme depends on its specific cellular localization and molecular substrate. The involvement of several HDACs in brain disorders has already been described (Simões-Pires et al., 2013; Volmar and Wahlestedt, 2015).

Among all the HDAC enzymes, HDAC6 has emerged as a possible target for disease modification in neurodegenerative diseases over the past few years (Li et al., 2011; Simões-Pires et al., 2013; Van Helleputte et al., 2014). This particular interest in HDAC6 appears to be related to its structural and functional characteristics that make HDAC6 unique compared to other classical HDAC enzymes (Yang and Grégoire, 2005; Simões-Pires et al., 2013). For instance, HDAC6 is the only deacetylase with two functional N-terminal catalytic domains, a C-terminal zinc finger ubiquitin-binding domain, and a tetradecapeptide repeating domain, which together with two leucine-rich nuclear export sequences, contributes to the cytosolic expression of HDAC6 (Li et al., 2011). Such properties allow HDAC6 to interact mainly with cytoplasmic substrates, with particular functions in cellular signaling systems involving not only acetylation but also ubiquitination of proteins and consequent protein degradation (Zhang et al., 2003; Lee et al., 2010). This protein drew even more attention among the research community upon the discovery that it was identified as a component of several pathological inclusions associated with AD, PD, and MSA (Ding et al., 2008; Miki et al., 2011; Chiba et al., 2012). In post-mitotic cells, efficient protein degradation is a crucial step to maintain cellular homeostasis, however, the precise implications of HDAC6 as a therapeutic target in these neurodegenerative disorders remain undefined.

## Structure and Functions of HDAC6

**Figure 1:**
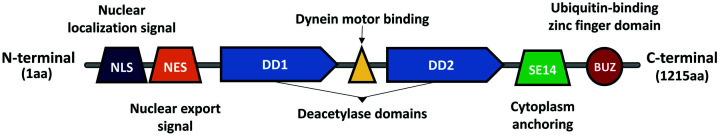
Histone deacetylase 6 (HDAC6) structure and domains. HDAC6 is a Class II histone deacetylase, composed of 1,215 amino acids. It is the only member from the Class II deacetylases containing two active catalytic domains (DD1 and DD2), which allow it to interact with different substrates (e.g., α-tubulin, HSP90, cortactin, et cetera). Due to the nuclear localization signal (NLS) and the nuclear export signal (NES), HDAC6 can shuttle between the nucleus and cytoplasm, however, the presence of a cytoplasm anchoring domain (SE14) ensures that HDAC6 is stably present in the cytoplasm. Between DD1 and DD2 exists a dynein motor binding domain, which allows HDAC6 to interact with the dynein motor complex and modulate cargo trafficking alongside the microtubules. In the C-terminal exists a ubiquitin-binding zinc finger domain (BUZ) with a high affinity to poly-ubiquitinated protein chains.

**Figure 2:**
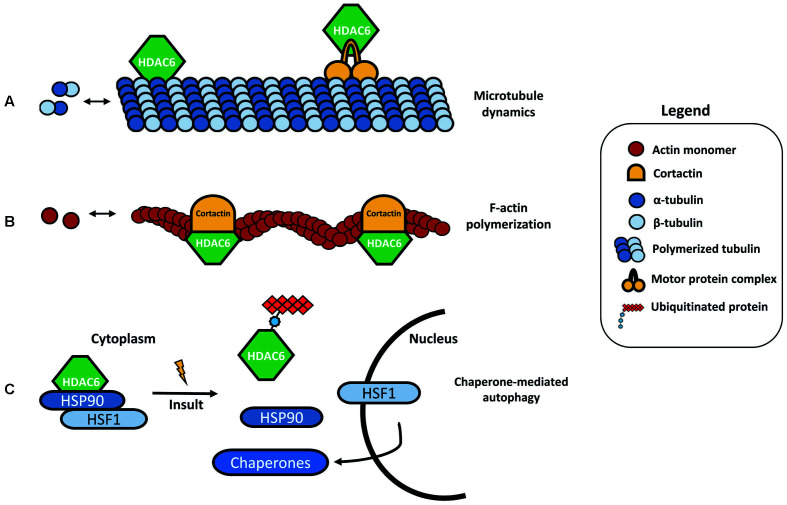
HDAC6 substrates. **(A)** One of the most studied targets of HDAC6 deacetylation is α-tubulin, an important subunit from the microtubules network. By binding to α-tubulin and inducing its deacetylation, HDAC6 can modulate the microtubule dynamics and cell motility. HDAC6 is also able to bind specific protein motor complexes, thus interfering with protein and cargo trafficking. **(B)** Cortactin is also a target for HDAC6 deacetylation. The interaction between HDAC6 and cortactin can modulate actin polymerization. **(C)** HSP90 under basal conditions forms a complex with HDAC6 and heat-shock transcription factor 1 (HSF1), however, in the presence of ubiquitinated protein aggregates, HDAC6 dissociates from this complex, leading to HSF1 activation and translocation to the nucleus to trigger the expression of major cellular chaperones involved in chaperone-mediated autophagy (CMA).

**Figure 3:**
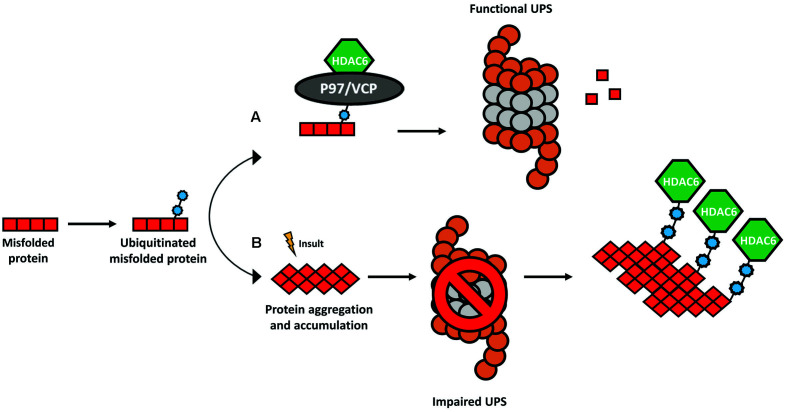
The ubiquitin-binding activity of HDAC6. The presence of a ubiquitin-binding domain in HDAC6 allows it to interact with higher affinity to poly-ubiquitinated protein chains. **(A)** Under physiological conditions, HDAC6 and the chaperone-like P97/VCP constitute a complex whereas an excess of P97/VCP over HDAC6 facilitates the release of ubiquitinated proteins for degradation in the proteasome. **(B)** In several neurodegenerative disorders, the formation of protein aggregates leads to a dysfunctional ubiquitin-proteasome system (UPS) and consequent accumulation of poly-ubiquitinated proteins. HDAC6 senses the increase in poly-ubiquitin chains and binds to them with higher affinity coordinating an alternative cellular protein degradation pathway.

HDAC6 was identified in 1999 as a protein composed of 1215 amino acids and the only HDAC enzyme containing a full duplication of Class I and II HDAC-homology domain (Grozinger et al., 1999; Verdel and Khochbin, 1999). It contains two active catalytic domains (DD1 and DD2), a ubiquitin zinc-dependent C-terminus domain and a conserved nuclear export signal (NES) at the N-terminus, responsible for the main expression of HDAC6 in the cytoplasm of different cell types (Bertos et al., 2004; Hai and Christianson, 2016; Figure 1). According to the literature, the implications of both catalytic domains in the deacetylase activity of HDAC6 are not well understood. Some studies report that both domains are needed for the deacetylation of α-tubulin, whereas others show that the inhibition of DD2 catalytic domain results in less α-tubulin deacetylation, suggesting that the second catalytic domain is responsible for the deacetylation of tubulin substrates (Haggarty et al., 2003; Zhang et al., 2006). Due to its unique structure and localization, HDAC6 is allowed to interact with a wide number of cytoplasmic substrates (Li et al., 2013). The most studied HDAC6 targets are α-tubulin, cortactin, and HSP90 (Hubbert et al., 2002; Zhang et al., 2003, 2007; Kovacs et al., 2005; Figure 2). The deacetylation of α-tubulin and cortactin makes HDAC6 an important regulator of the microtubule and actin cytoskeleton dynamics, respectively, contributing to the control of cell motility, adhesion and intracellular cargo trafficking (Zhang et al., 2007; Zilberman et al., 2009; Chen et al., 2010; Figures 2A,B). By promoting the deacetylation of HSP90, HDAC6 appears to have an important role in one of the main cellular mechanisms to cope with misfolded proteins (Boyault et al., 2007b; Figure 2C). Also, due to the ubiquitin zinc-dependent domain located at the C-terminal, HDAC6 can bind with high affinity to ubiquitinated proteins suggesting a pivotal role of HDAC6 in another cellular proteolysis mechanism (Boyault et al., 2006; Figure 3). Such features put HDAC6 on the list of therapeutic targets in neurodegenerative diseases, in which protein accumulation and aggregation leads to neurodegeneration in different regions of the brain.

## HDAC6 Interacting Partners and Specific Role in Protein Degradation

Newly synthesized proteins need the proper biological machinery to adopt the correct conformation. During this process, some proteins can incorrectly fold and form larger aggregates (Balchin et al., 2016). In turn, such aggregates are harmful and constitute one of the common features of neurodegenerative disorders (Ross and Poirier, 2004). Therefore, neuronal cells depend highly on proteolysis and other protein quality control mechanisms to maintain homeostasis in the CNS. As mentioned above, HDAC6 is an important player in several biological processes and it was observed that it is a key regulator of three cellular mechanisms triggered to cope with the accumulation of protein aggregates: (1) the formation of the aggresome and consequent autophagy; (2) the binding to poly-ubiquitinated chains of misfolded proteins; and (3) the deacetylation of HSP90. These mechanisms rely on HDAC6 either through its deacetylase activity, or ubiquitin-binding properties.

HDAC6 is involved in the regulation of the UPS, a cellular mechanism for the degradation of small, soluble, and non-aggregated proteins (Ciechanover and Brundin, 2003). Under physiological conditions, HDAC6 forms a complex with a chaperone-like 97 kDa p97/VCP protein, an adenosine triphosphatase with a pivotal role to transfer ubiquitinated proteins to the proteasome (Boyault et al., 2006; Figure 3A). In neurodegenerative disorders, the UPS is impaired, and consequently, poly-ubiquitin protein chains accumulate (Bence et al., 2001). HDAC6 binds the polyubiquitinated proteins to mediate the formation of the aggresome and enable alternative protein degradation through the autophagy-lysosome pathway (Pandey et al., 2007; Figure 3B).

**Figure 4:**
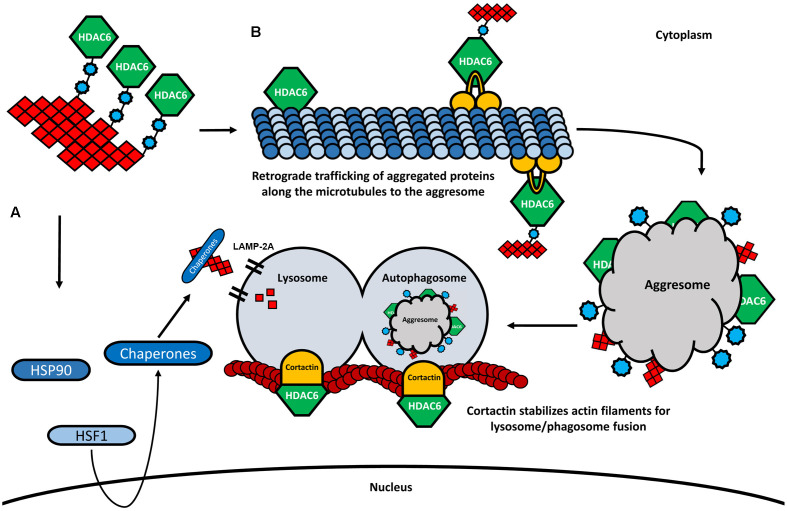
HDAC6 and its involvement in CMA and aggresome formation. **(A)** In the presence of higher amounts of ubiquitinated proteins, HDAC6 dissociates from the HSP90/HSF1 complex, leading to the activation of heat-shock response and degradation of aggregated proteins by the lysosome *via* LAMP-2A. **(B)** HDAC6 binds to ubiquitinated proteins through its ubiquitin-binding domain. Also, HDAC6 interacts with the motor protein dynein and retrogradely transports the aggregated proteins along the microtubules to the perinuclear region where the aggresome is formed. The aggresome is then transferred to the autophagosome, which is fused to the lysosome. The fusion of the autophagosome and the lysosome is further modulated by HDAC6 through the deacetylation of cortactin.

Under stress conditions, the activation of a heat-shock gene response complex is triggered, leading to the expression of several chaperones. The activity of the chaperones prevents the aggregate formation by assisting the delivery of misfolded proteins to the UPS, or by mediating the chaperone-mediated autophagy (CMA; Waza et al., 2006). One of these chaperones is the HSP90, another deacetylase target of HDAC6 (Kovacs et al., 2005). HSP90 forms a complex with HDAC6 and the heat-shock transcription factor 1 (HSF1), which remains inactive in normal conditions (Boyault et al., 2007b; Figure 4A). Again, the increased levels of ubiquitination will favor the deacetylation of HSP90 by HDAC6, leading to the dissociation of the complex. As a consequence, HSF1 is activated, induces the transcription of heat-shock proteins and co-chaperones, and initiates a process of CMA (Boyault et al., 2007b).

As already mentioned, one of the most studied deacetylase targets of HDAC6 is α-tubulin, an important subunit of microtubules. Acetylated α-tubulin plays an important role in axonal trafficking and cargo transfer, by promoting the interaction between the motor proteins dynein, kinesin-1, and the microtubule network, therefore interfering with both anterograde and retrograde trafficking of cargo (Reed et al., 2006; Figure 4B). For example, velocity and motility of the mitochondria and the transport of proteins have been shown to depend on both anterograde and retrograde axonal transports modulated by HDAC6 inhibition and tubulin acetylation (Bulinski, 2007; Dompierre et al., 2007; Chen et al., 2010; Kim et al., 2012). The formation of the aggresome and consequent autophagic clearance of protein aggregates also depends on the deacetylase activity of HDAC6 and the ability to bind poly-ubiquitinated protein chains (Figure 4B). Through the interaction with both ubiquitinated proteins and with the dynein motor complex, HDAC6 mediates the transfer of protein aggregates towards the microtubule organizing center (MTOC), the region where the aggresome formation takes place (Johnston et al., 1998; Kopito, 2000; Kawaguchi et al., 2003). Indeed, the knock-down of HDAC6 resulted in an impairment of the aggresome formation and increased apoptosis in cultured cells, suggesting an important role of the HDAC6-ubiquitin-dynein complex for the transportation of aggregated proteins and the formation of the aggresome (Kawaguchi et al., 2003; Figure 4B). Subsequently, lysosome-dependent macroautophagy is triggered to degrade the aggregated proteins of the aggresome (Fortun et al., 2003; Chin et al., 2010). It is believed that the autophagic machinery is recruited to the aggresome in the same way that substrates are accumulated at the MTOC (Iwata et al., 2005). Interestingly, by promoting the reorganization of the actin filaments through the deacetylation of cortactin, HDAC6 plays also an important role facilitating the fusion of lysosomes with autophagosomes, where the aggregates are degraded (Lee et al., 2010; Wang et al., 2018; Figure 4B). The depletion of HDAC6 prevents cortactin deacetylation, resulting in impaired lysosome-autophagosome fusion and consequent decrease of autophagic activity (Lee et al., 2010).

These observations suggest a decisive role of HDAC6 in the initiation of three main protein degradation mechanisms.

## Involvement of HDAC6 in Neurodegenerative Disorders

The involvement of HDAC6 in several neurodegenerative disorders has already been described, however, the functions of HDAC6 in the progression of neurodegeneration are not yet understood.

In AD, HDAC6 is significantly increased in the hippocampus and other relevant brain regions both in AD patients and in models of the disease (Zhang et al., 2013). However, the role of HDAC6 in the progression of AD pathology is still unclear. Some studies report that HDAC6 has a beneficial effect by rescuing neurodegeneration while others believe that HDAC6 contributes to AD-associated neurodegeneration. One example of the detrimental role of HDAC6 associated with AD is the direct interaction with the microtubule-associated protein tau (Ding et al., 2008). *In vivo* and *in vitro* experiments show that HDAC6 can bind to tau and modulate its phosphorylation leading to the formation of neurofibrillary tau tangles (Ding et al., 2008; Cook et al., 2012, 2014). In contrast, HDAC6 can also rescue neurodegeneration by participating in the formation of tau-containing aggresomes. Proteasome inhibition *in vitro* resulted in the tau aggresome formation mediated by the ubiquitin-binding activity of HDAC6 (Guthrie and Kraemer, 2011). Therefore, HDAC6 in AD pathology may play a dual role: on one hand, it mediates the hyperphosphorylation of tau and formation of neurofibrillary tangles; on the other hand, it enables the formation of the aggresome and triggers alternative protein degradation by the autophagy-lysosomal pathway.

Huntington’s disease (HD) is characterized by the accumulation of the huntingtin protein (htt) and disturbances in axonal transport, resulting in cognitive decline, dementia, and impairment in motor coordination (Walker, 2007). Also, microtubule-dependent transport and tubulin acetylation are decreased in HD, leading to impaired transport of neurotrophic factors and cell death (Gunawardena et al., 2003). The use of HDAC6 inhibitors in a cellular model of HD increased acetylated tubulin and enhanced the recruitment of the dynein motor complex to the microtubules, stimulating the transfer of brain-derived neurotrophic factor (BDNF), in the cells (Dompierre et al., 2007). Furthermore, HDAC6 appeared to have an important role in the degradation of htt by recruiting the autophagic machinery to inclusion bodies containing htt in a neuronal cell model of HD (Iwata et al., 2005). However, in a mouse of HD, the inhibition of HDAC6 showed contradictory effects. Despite increased levels of tubulin acetylation, the authors observed no effects on BDNF transport, nor on the levels of soluble mutant htt (Bobrowska et al., 2011).

HDAC6 is associated with familial cases of amyotrophic lateral sclerosis (ALS), in which mutations in the DNA binding protein TDP-43 result in impaired microtubule-dependent axonal trafficking of mRNAs and the formation of TDP-43 aggregates. Interestingly, TDP-43 can bind to HDAC6 mRNA, regulating its expression (Fiesel et al., 2011). Also, mutations in the chaperone-like p97/VCP are found in ALS cases, resulting in the accumulation of ubiquitin-positive aggregates found in ALS patients (Johnson et al., 2010). The interaction between HDAC6 and p97/VCP is known to orchestrate the degradation of ubiquitinated proteins through the UPS, suggesting a possible role of HDAC6 in ALS pathogenesis (Van Helleputte et al., 2014). It was observed that blocking HDAC6 activity increased insoluble TDP-43 levels *in vitro*, suggesting that HDAC6 inhibition may exacerbate TDP-43 accumulation (Chen and Cohen, 2019).

## The Role of HDAC6 in α-Synucleinopathies

The association between HDAC6 and the pathogenesis of α-Synucleinopathies emerged when histopathological analysis in brain sections from PD patients revealed highly concentrated expression of HDAC6 within LBs, suggesting a possible role of HDAC6 in the formation of such inclusions (Kawaguchi et al., 2003; Miki et al., 2011). Besides, a recent study showed that not only HDAC6 but also its phosphorylated form was co-localized with α-Syn in inclusions derived from postmortem brains of PD and MSA patients (Mazzetti et al., 2020). Despite the small cohort of patients used in this study, the presence of phosphorylated HDAC6 seems to be a common feature of intracellular inclusions in α-Synucleinopathies, while it was absent in β-amyloid plaques derived from AD brains. The location of α-Syn inclusions in the brain of patients with PD and MSA is not only limited to the cytoplasm and can be found in the nucleus of neuronal and glial cells. Indeed, several studies have reported that epigenetic disturbances and dysregulation in histone deacetylation play a role in the pathogenesis of PD (Mazzocchi et al., 2020). The presence of α-Syn in the nucleus was shown to interfere with histone acetylation, resulting in gene expression alterations and neurotoxicity *in vitro* (Goers et al., 2003; Kontopoulos et al., 2006). Such neurotoxic effects were rescued upon the treatment with an HDAC inhibitor (Kontopoulos et al., 2006). Another study in a mouse model of MSA showed that histone deacetylation inhibition presented a neuroprotective role in the pathogenesis of MSA-like neurodegeneration (Sturm et al., 2016).

Although there are substantial findings regarding the potential neuroprotective effects in the modulation of HDACs activity in α-Synucleinopathies, the focus on HDAC6 increased with the demonstration that HDAC6 plays an important role in the regulation of different protein degradation mechanisms. As mentioned above, HDAC6 plays a pivotal role in the formation of the aggresome, a cellular defense mechanism to cope with higher amounts of misfolded proteins (Kawaguchi et al., 2003). Interestingly, LBs and aggresomes share important biochemical and morphological properties that led researchers to hypothesize that the formation of LBs is an aggresome-related process (Olanow et al., 2004; Tanaka et al., 2004). Indeed, proteins associated with the formation of the aggresome were found in LBs from PD and DLB patients (McNaught et al., 2002). Besides, LBs can sequester heat-shock proteins such as HSP70, together with proteins associated with the UPS system, also found in the aggresome to enhance protein degradation (Lee and Lee, 2002; McNaught et al., 2002).

Intriguingly, the involvement of HDAC6 in the pathogenesis of PD has been further supported in genetic cases. *ATP13A2*, a gene that is mutated in autosomal recessive juvenile PD, was experimentally shown to facilitate HDAC6 recruitment to the lysosome leading to disruption of autophagosome-lysosome fusion by increasing the cortactin acetylation (Wang et al., 2019).

In oligodendrocytes, the presence of aggresome-related proteins together with HDAC6 were found also co-localized with GCIs derived from brain samples of MSA patients, suggesting that oligodendrocytes may use the same aggresome machinery for the formation of such MSA-specific α-Syn inclusions (Chiba et al., 2012). However, GCIs and aggresomes present some differences. For instance, one important step for the aggresome formation is the redistribution of vimentin, an intermediate filament protein, forming a cage-like structure surrounding aggregated and ubiquitinated proteins in the perinuclear region (Johnston et al., 1998). According to the literature, mature oligodendrocytes are devoid of intermediate filament networks, suggesting that the presence of other cytoskeleton proteins may be involved in the formation of GCIs in oligodendrocytes (Chiba et al., 2012; Kaji et al., 2020). Taking this into consideration as well as the involvement of HDAC6 in different protein quality control mechanisms prompted researchers to investigate whether HDAC6 could be involved in the formation of α-Syn containing inclusions.

Therefore, further studies focused on the role of HDAC6 in α-Synucleinopathy models (Table 1). In general, the reported data have shown some controversies that may be related to the use of different models and different methods for HDAC6 modulation. Several studies suggested that HDAC6 deficit induced by either pharmacological inhibition (Tubastatin A, Tubacin, Trichostatin A) or genetic modification (knockout, siRNA, shRNA) leads to an increase of oligomeric α-Syn commonly associated with lower cell viability and reduction of the intracellular inclusion burden. These effects have been attributed to impaired aggresome formation and disruption of autophagy mechanisms (Du et al., 2010, 2014; Su et al., 2011; Ejlerskov et al., 2013). On the other hand, Francelle et al. (2020) reported that HDAC6 inhibition in an AAV-α-Syn overexpression model resulted in higher levels of HSP70 and LAMP-2A linked to lower α-syn phosphorylation suggesting a leading role of CMA in this setting, finally leading to the neuroprotection of dopaminergic neurons. Alternatively, HDAC6 overexpression was suggested to lower α-Syn oligomer levels but substantially increase the level of insoluble protein and inclusion formation, which the authors related to reduced cellular toxicity (Du et al., 2010, 2014). Importantly, the Class III HDAC, Sirtuin 2, expressed also in the cytoplasm, has been associated with the modulation of tubulin acetylation and its inhibition led to neuroprotection linked to enlargement of α-Syn aggregates in PD models (Outeiro et al., 2007). Whether HDAC6 and Sirtuin 2 may share common mechanisms of neuroprotection in α-Synucleinopathy is not completely clear. Recently, Mazzocchi and collaborators (Mazzocchi et al., 2020) proposed that the neuroprotective effects of HDAC6 may be due to the modulation of the proteinopathy, however, rescue effects may depend on the cellular context, e.g., linked to other processes dependent on the cytoskeletal dynamics like axonal transport of synaptic activity. Further studies will be needed to understand the interference of HDAC6 with α-Syn toxicity concerning its structure and interaction partners, which may tune the interaction outcome in the different experimental systems.

**Table 1:** Current evidence on the effects of HDAC6 modulation on α-Syn aggregation.

Reference	α-Synucleinopathy model	HDAC6 modulation strategy	Effects on α-syn pathology
Du et al. (2010)	Human α-Syn expression in *Drosophila*	Knockout	• Reduced number of α-Syn inclusions • Increased levels of α-Syn oligomers
		Overexpression	• Increased number of α-Syn inclusions • Decreased levels of α-Syn oligomers
Su et al. (2011)	PC12 and SH-SY5Y cell lines with A53T mutant α-Syn overexpression + MPP+ treatment	siRNA Tubacin A	• Intracellular accumulation of α-Syn without the formation of inclusions
			• Increased levels of α-Syn in the nucleus
Ejlerskov et al. (2013)	The differentiated PC12 cell line with mutant A30P α-Syn overexpression + TPPP/p25α expression	shRNA Trichostatin A	• p25α inhibition of HDAC6 activity resulting in impaired autophagosome maturation and consequent α-Syn exophagy.
Du et al. (2014)	Lactacystin UPS-impairment in mice	Trichostatin A	• Increased α-Syn oligomers in SNc
	SK-N-SH neuroblastoma cell line with α-Syn overexpression	Knockdown	• Increased α-Syn oligomers levels
		Overexpression	• Decreased α-Syn oligomers levels • Increased α-Syn inclusion formation
Francelle et al. (2020)	AAV-α-Syn rat model	Tubastatin A	• Activation of autophagy pathways • Decreased α-Syn phosphorylation

It is currently unclear whether the interaction between α-Syn and HDAC6 may be bidirectional. It was previously proposed that α-Syn in the nucleus may interfere with histone acetylation and HDAC inhibition may rescue the pathology in cell culture and in flies (Kontopoulos et al., 2006). Similar effects of α-Syn in the cytoplasm on the acetylation of other proteins have not been reported yet.

## Concluding Remarks

To summarize, it becomes clear that HDAC6 is involved in the pathology of α-Synucleinopathies by interfering with the accumulation of α-Syn oligomers and the formation of protein aggregates. Importantly, LBs in neurons and GCIs in oligodendrocytes differ in the structure supporting the idea of a different origin and/or trigger of α-Syn aggregation. However, LBs and GCIs share common components including HDAC6, ubiquitin, and heat shock proteins. This fact suggests that the same protein clearance mechanisms may act to rescue the cells from the accumulating misfolded α-Syn, however remaining inefficient.

HDAC6 may represent an attractive target for the therapy of α-Synucleinopathies, however, the effects of its enzymatic (deacetylation activity) or non-enzymatic activity (binding to ubiquitin) on α-Syn inclusion formation are still not conclusive. Models, selectively modulating each of these activities may help decipher their involvement and roles in the pathogenesis of α-Synucleinopathies. Understanding the role of possible interaction partners may be crucial for the interpretation of the outcomes of HDAC6 modulation in the variable experimental systems. Finally, understanding how the effects of HDAC6 on other basic cellular mechanisms may interfere with the vulnerability to α-Syn will be crucial in the future search for strategies to target HDAC6 for disease modification in α-Synucleinopathies.

## Author Contributions

ML wrote the first draft of the manuscript. NS conceived the idea and made revisions to the text. Both authors have seen and approved the submitted version.

## Conflict of Interest

The authors declare that the research was conducted in the absence of any commercial or financial relationships that could be construed as a potential conflict of interest.

## Abbreviations

AD: Alzheimer’s disease
ALS: amyotrophic lateral sclerosis
BDNF: brain-derived neurotrophic factor
CMA: chaperone-mediated autophagy
CNS: central nervous system
DD1: and DD2 HDAC6 deacetylase domain 1 and 2
DLB: dementia with Lewy bodies
GCIs: glial cytoplasmic inclusions
HAT: histone acetyltransferases
HD: Huntington’s disease
HDAC6: histone deacetylase 6
HSF1: heat-shock transcription factor 1
HSP90: heat-shock protein 90
Htt: huntingtin protein
LBs: Lewy bodies
LNs: Lewy neurites
MPP+: 1-methyl-4-phenylpyridinium
MSA: multiple system atrophy
MTOC: microtubule-organizing center
NAC: non-Amyloid β component
PD: Parkinson’s disease
SNc: substantia nigra pars compacta
TDP-43: TAR DNA-binding protein 43
TPPP/p25α: Tubulin polymerization-promoting protein
UPS: ubiquitin-proteasome system
α-Syn: α-Synuclein.
